# Creating PWMs of transcription factors using 3D structure-based computation of protein-DNA free binding energies

**DOI:** 10.1186/1471-2105-11-225

**Published:** 2010-05-03

**Authors:** Denitsa Alamanova, Philip Stegmaier, Alexander Kel

**Affiliations:** 1 BIOBASE GmbH, Halchtersche Strasse 33, D-38304 Wolfenbuettel, Germany

## Abstract

**Background:**

Knowledge of transcription factor-DNA binding patterns is crucial for understanding gene transcription. Numerous DNA-binding proteins are annotated as transcription factors in the literature, however, for many of them the corresponding DNA-binding motifs remain uncharacterized.

**Results:**

The position weight matrices (PWMs) of transcription factors from different structural classes have been determined using a knowledge-based statistical potential. The scoring function calibrated against crystallographic data on protein-DNA contacts recovered PWMs of various members of widely studied transcription factor families such as p53 and NF-*κ*B. Where it was possible, extensive comparison to experimental binding affinity data and other physical models was made. Although the p50p50, p50RelB, and p50p65 dimers belong to the same family, particular differences in their PWMs were detected, thereby suggesting possibly different in vivo binding modes. The PWMs of p63 and p73 were computed on the basis of homology modeling and their performance was studied using upstream sequences of 85 p53/p73-regulated human genes. Interestingly, about half of the p63 and p73 hits reported by the Match algorithm in the altogether 126 promoters lay more than 2 kb upstream of the corresponding transcription start sites, which deviates from the common assumption that most regulatory sites are located more proximal to the TSS. The fact that in most of the cases the binding sites of p63 and p73 did not overlap with the p53 sites suggests that p63 and p73 could influence the p53 transcriptional activity cooperatively. The newly computed p50p50 PWM recovered 5 more experimental binding sites than the corresponding TRANSFAC matrix, while both PWMs showed comparable receiver operator characteristics.

**Conclusions:**

A novel algorithm was developed to calculate position weight matrices from protein-DNA complex structures. The proposed algorithm was extensively validated against experimental data. The method was further combined with Homology Modeling to obtain PWMs of factors for which crystallographic complexes with DNA are not yet available. The performance of PWMs obtained in this work in comparison to traditionally constructed matrices demonstrates that the structure-based approach presents a promising alternative to experimental determination of transcription factor binding properties.

## Background

The binding affinities of transcription factors (TFs) to short DNA sequences play a major role in the gene regulation and thus in the proper functioning of the cell machinery. Understanding the mechanisms of TF binding specificity has become an important goal for theoreticians and experimentalists. With the development of novel experimental techniques like ChIP-Chip and ChIP-Seq, covering TF binding over the whole genome, the need of unbiased theoretical methods recovering the binding event has grown immensely. Along with the classical approaches like Molecular Dynamics [1] (MD) and Monte Carlo [2] (MC) that place high demands on computer resources several knowledge-based potentials [3-6] have been developed for calculation of protein-DNA binding energies. Simulations in the course of MD and MC are dependent on the quality of the input protein and DNA structures, which are often taken from the Protein Data Bank (PDB) [7]. Another complication is the high flexibility of the DNA-binding domain and sometimes also the ligand itself. An alternative approach that most often does not consider the molecular flexibility is docking. The scoring functions applied in docking methods basically contain electrostatic and van der Waals components [8] but could be extended to complicated free energy models that also take into account solvation effects and hydrogen bond direction [5].

However, for a given DNA sequence of length *N*, there are altogether 4^*N *^variants the TF could bind, which for moderately long motifs containing 18-21 bases grow to computationally infeasible number. In the last years, much effort has been devoted to the development of knowledge-based potentials [9] that take into account 3D crystal structures of known TF-DNA complexes for estimation of parameters of such potentials. Using such potentials one can predict binding energy of a given TF to new unknown target DNA sequence. On the other hand, traditionally, prediction of TF binding sites in DNA sequences is often done with the help of so called position weight matrices (PWMs), that represent a simple model of TF-DNA binding in a form of a 4 × N matrix [10]. A similarity score can be computed for any DNA sequence of length N according to a PWM. A specific threshold for the score is defined, to classify sequence segments as binding sites for corresponding transcription factor(s). Compilations of such PWMs for many transcription factors are represented in databases, such as TRANSFAC [11] and Jaspar [12]. Normally, the PWMs collected there are constructed from alignments of known, experimentally proven TF binding sites obtained by gel-shift analysis, SELEX, plasmid construction assays and other experimental techniques. A possible bias and inaccuracy of such PWMs could result from predominant inclusion of strong and relatively strong binding sites as most experiments could not detect lower-affinity sites. Knowledge-based potential can help to improve detection of such low-affinity sites through the use of information about the contact frequencies between different residues or atoms in known crystal structures to predict the interaction energy. Here we present a new method for constructing PWMs using a knowledge-based potential. For a TF of interest, our method performs computational estimation of the relative contribution of each nucleotide of the DNA sequence to the free energy of binding.

In the present work a statistical knowledge-based potential [3] was adopted for studying the free binding energies to DNA of several TFs from different classes. We chose this potential as it outperformed other knowledge-based potentials in discriminating the native structure from other near-native decoys. As we investigated not only crystal structures of protein-DNA complexes but also had to apply homology modeling in order to obtain complexes for which crystallographic structures had not been resolved, this turned out to be a major reason for the choice of this potential. For most of the TFs studied here we found in the literature experimental data on their binding affinity to various DNA target sequences. Especially the tumour suppressor p53 and its interaction with DNA has been extensively studied. Its role in cell cycle arrest or apoptosis and affinity for specific DNA binding sites make it a suitable candidate for theoretical and experimental studies. As its resolved crystal structure contains four monomers bound to the DNA chain, it is computationally difficult to derive the corresponding PWM using molecular dynamics methods. Moreover, many proteins are commonly found in their homodimeric form in the bound state. Another typical problem is the availability of protein-DNA crystal complexes. For example, crystal structures of p63 and p73 in a complex with DNA have not been resolved, but solution structures of their DNA-binding domains (DBDs) are known. Both p63 and p73 reach 63% amino acid similarity to p53 in the DBD, suggesting that their PWMs could be slightly different from the p53 PWM. As in TRANSFAC there are no specifically constructed PWMs for each of these p53 family members, adequate PWMs could be derived using homology modeling combined with a statistical potential taking into account atomistic details.

We compared the tetramer p53 PWM presented here to available experimental data and other theoretical approaches and the good agreement encouraged us to calculate PWMs of proteins from different TF classes that have been experimentally studied, such as the NF-*κ*B family, GABP and ER*α*. Finally, we evaluated the performance of the newly computed PWMs of p53- and NF-*κ*B-family members in detecting binding sites in known target DNA sequences derived through ChIP experiments.

## Methods

### Statistical potential

For the estimation of the protein-DNA interaction energy we used the all-atom statistical potential developed by Robertson and Varani [3] that was successfully applied in discrimination of near-native structures from docking decoys. This distant-dependent potential was constructed using crystallographic data from native protein-DNA complexes. Briefly, the free energy of the complexes was mimicked by a thermodynamical potential function that took into account the protein-DNA interface contact distances and the chemical atom types. The protein and nucleic acid heavy atom types were considered in a residue-specific manner. The probability of an interatomic contact was expressed in terms of the likelihood of observing a particular distance between a protein and a DNA atom in a native-like, 'correct' complex. The logarithm of this probability of correctness *P*(*C*|*D*) of the interatomic distances described the Gibbs free energy of the complex and was negative for native-like complexes:(1)

In the formula, *D *is the set of atomic distances *d*_*ij *_between the interface atoms, *t*_*i *_and *t*_*j *_correspond to the chemical types of the atoms, *N*_*P *_and *N*_*D *_represent the number of protein and DNA atoms in the complex. The probability of an individual atomic contact is modeled as the likelihood of observation of a separation *d*_*ij *_between atoms *t*_*i *_and *t*_*j *_in a native-like protein-DNA complex:(2)

where *P*(*C*|*d*_*ij*_, *t*_*i*_, *t*_*j*_) is the likelihood function, *P *(*d*_*ij*_, *t*_*i*_, *t*_*j*_) the marginal probability, and *P*(*C*) the Bayesian prior representing the probability of observing a native-like protein-DNA complex. Due to difficulties in determination of the value of *P*(*C*), it was set to one in [3]. Finally, the likelihood of observation a native-like interatomic distance *d*_*ij *_was expressed with the formula:(3)

where *N*_*obs*_(*d*_*ij*_, *t*_*i*_, *t*_*j*_) is the number of contacts observed between two atoms of type *t*_*i *_and *t*_*j *_separated by distance *d*_*ij*_. This statistical function maps the continuous value *d*_*ij *_to a set of discrete distance bins, the atoms separated by distances greater than or equal to *d*_*ij *_are not counted. In the present work the 3/10/1 range of the potential was used, where the scoring function considers all interface protein and DNA atoms separated by less than 10Å, and groups these contacts into eight bins beginning with a 3Å bin, followed by seven 1Å bins. It has been shown [3] that a larger distance cutoff would not improve significantly the results, under certain circumstances it could rather lead to false positives, hence in the present study the 3/10/1 parameters were used.

### Computation of the PWMs

For the estimation of protein-DNA free binding energies structures of the transcription factors cocrystallized with DNA were used. The crystal structure of entry 2AC0 from the PDB databank [7] was used for the calculation of the p53 tetramer PWM, for the homodimer the complex with entry 2GEQ was selected. We calculated also the PWMs of three members of the NF-*κ*B family: the p50 homodimer (PDB entry 1NFK), p50RelB (2V2T), and the p50p65 heterodimer (1VKX). Finally, the heterodimeric complex of GABP (PDB code 1AWC) and the homodimeric ER*α *cocrystallized with DNA (PDB code 1HCQ) were used as input for the calculations of the corresponding PWMs. In the cases where more than one crystal structure of the TF complexed with DNA was available, as in the case of 1VKX, a custom script was used to estimate the van der Waals energy arising from the TF and DNA interatomic contacts. The lower van der Waals repulsion energy, as in the case of the 1VKX structure, guarantees that the number of 'bad', unnatural TF-DNA atomic contacts occurring in the particular crystal structure is smaller than in the complexes showing higher van der Waals energy. Consecutively, complexes with the most native TF-DNA interatomic distances will provide the most correct TF-DNA free binding energies in the scoring procedure.

The crystal structures of the DNA chains taken from the corresponding TF-DNA complexes were mutated using the MMTSB (Multiscale Modeling Tools for Structural Biology) [13] script mutateNA.pl by fixing the chain backbone and substituting one base pair at each step. Sterical inaccuracies were avoided as the script used a library of torsion parameters for the correct residue rotations. For example, the calculated root mean square deviation (RMSD) between a 10 bp crystal DNA fragment and its completely mutated version was as small as 0.283Å when superimposing the C4 atoms.

**Figure 1:**
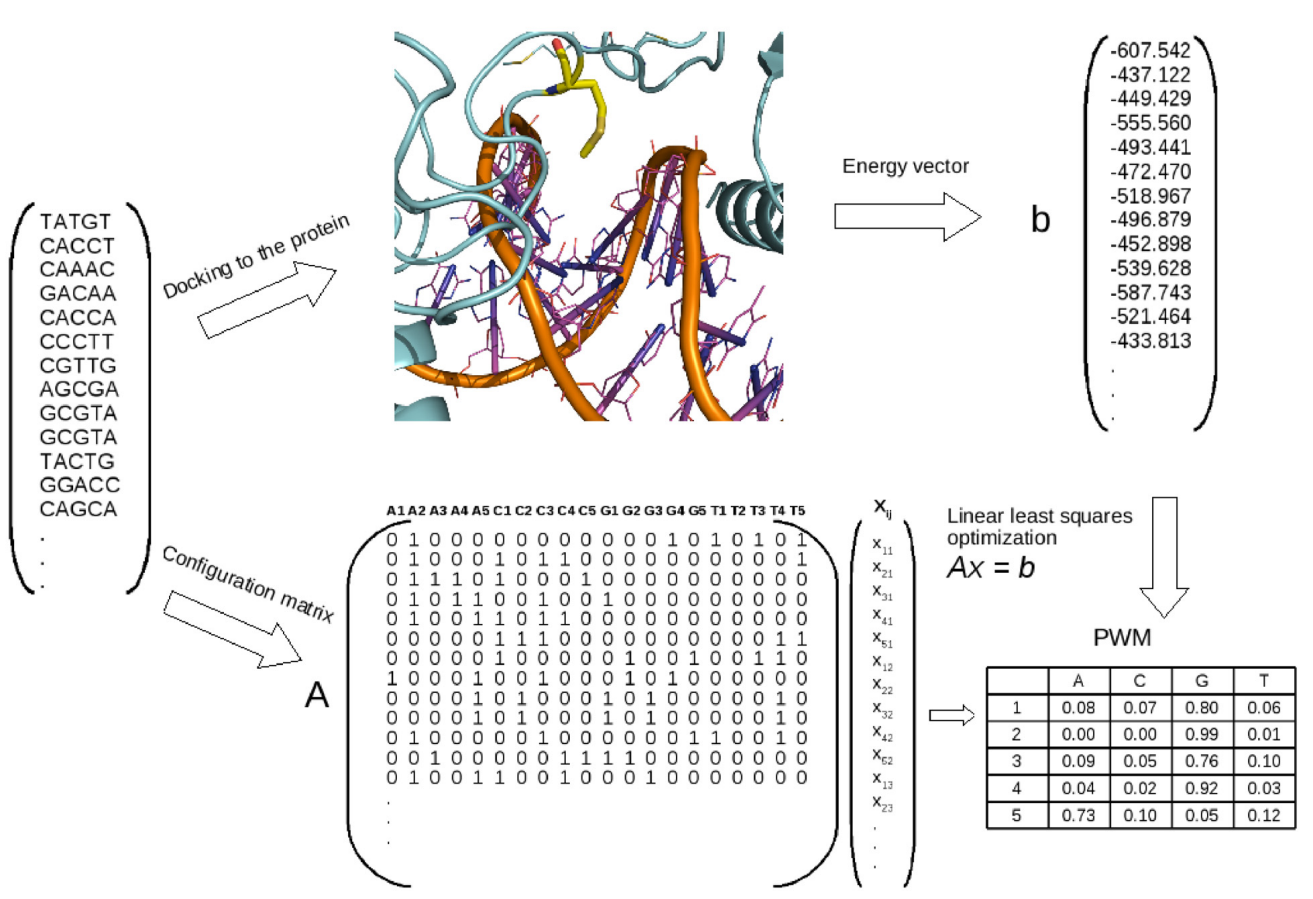
**The PWM computational scheme used in the present study**.

The workflow of structure-based PWM calculation developed in this study is schematically presented in Figure 1 and proceeds as follows: A 3D structure of a transcription factor bound to its target DNA sequence is retrieved from the PDB databank. For each DNA sequence of length *N *as found in the corresponding crystal structure we generate 4*N *+*X *random sequence fragments of the same length, where *X *could range between 1 and some large number, for computational efficacy we constrained this parameter to maximally 100 000 random sequences. Typically, 4*N *independent parameters are enough for the solution of the same number linear equations, however, in this case the use of a very small set of *X *additional sequences or no such at all results in slightly different weights w(*i*, *u*) (see Eq. 5) and resulting PWM coefficients, respectively. These small discrepancies (numbers after the comma) arise from the residue rotations while substituting nucleotides in the crystal DNA structure before scoring the protein-DNA contacts. In order to test the effect of adding these additional sequences, we generated also 50 000 random sequences and scored their binding free energy to p53. We found that already a small number of additional DNA sequences, *X *between 20 and 50, was fairly enough for accurate estimation of the particular energy contribution from each nucleotide. Further, for each of the 4*N*+*X *random sequences we compute the free energy of binding to the TF using the statistical potential described above.

The task is now to estimate all weights w(*i*, *u*) in the PWM (*i*-position in the sequence ranging from 1 to *N*, *u *- nucleotides A, C, G, T), so, that the binding energy predicted by the PWM would maximally correlate with the energy computed with the statistical potential. We chose to estimate the weights w(*i*, *u*) by solving the linear equation:(4)

where *X *is a vector of 4*N *dimensions of the estimated weights, so the components of the vector *X *contain all weights of the respective PWM in the following consequent manner:(5)

and *A *is a binary matrix of dimensions (4*N*, 4*N *+*X*), which contains information on all random DNA sequences whose free binding energy was computed. Each line of the matrix *A *corresponds to one DNA sequence. It contains 1 if the respective nucleotide is found in the corresponding position of the sequence and 0 otherwise. The free binding energy vector *b *consists of 4*N *+*X *values obtained with the protein-DNA scoring procedure described above. Eq.4 is solved by least squares optimization in the Octave statistical package [14].

We set the Bayesian prior *P*(*C*) in Eq.2 to one according to Ref. [3]. Respectively, the energy weights computed here were rescaled to reproduce the TF-DNA binding energy in the units of kJ/mol, using experimentally obtained binding affinities for each factor from the literature. Finally, nucleotide probabilities  were calculated from the corresponding energy coefficients  using the Boltzmann formula:(6)

where  is the energy contribution from a particular nucleotide *α*, *β *= 1*/RT *is the inverse energy (*R *is the universal gas constant in *J*. [*mol*. *K*]^-1^, T is the absolute temperature in Kelvin) and *γ *= {A, C, G, T}. The calculation of 50 000 TF-DNA complexes using a standard Pentium CPU takes a couple of hours depending on the DNA fragment length. All PWMs used in this work, both calculated from structure and collected from previous studies, are available in Additional File 1.

The performance of the newly computed PWMs of the members of the p53 and NFKB families was tested on upstream sequences of human genes known to be regulated by those factors. All upstream DNA sequences used here were extracted from the TRANSPRO^® ^[15] database taking various promoter windows for thorough analysis. The Match™ algorithm included in TRANSFAC version 2009.2 was used for promoter scan. All TRANSFAC matrices used in the present study were extracted from the same version.

## Results

### p53 tetramer

**Figure 2:**
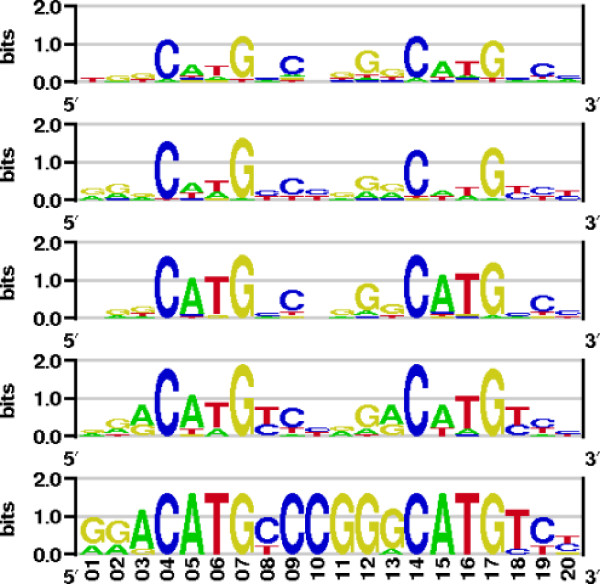
**Sequence logos for the p53 tetramer**. From top: the p53 tetramer PWM from this study, the experimental one from Ref. [19] derived from 100 sites, one computed with the DDNA2 server, experimental matrix from Ref. [18] obtained from affinity measurements, and the corresponding matrix V$P53_01 from TRANSFAC. All sequence logos presented here were made with enoLOGOS [39].

The tumor protein 53 is one of the experimentally and theoretically most extensively studied transcription factors, which makes it a very suitable system for validation of new methods for binding mode prediction. Here, the PWMs of its dimeric and tetrameric forms were calculated. The p53 tetramer response elements have two half-site palindromes with consensus sequence RRRCWWGYYY summing up to a 20 bp DNA sequence. Sequence logos of some of the p53 tetramer PWMs published in the last years together with the one reported here are shown in Figure 2. The third logo from the top was obtained using the DDNA2 server [16] that estimates the protein-DNA binding energy adopting a newly published version of the DFIRE potential [17]. In order to compare these five PWMs we estimated their relative entropies using the following formula:(7)

where *p*_*i*, *j *_is the probability of observing element *i *at position *j *in the PWM. Not surprisingly, both experimental PWMs have very similar relative entropies (3.22 from Ref. [18] and 3.99 for the PWM from Ref. [19]). From the theoretically predicted ones, the PWM reported here has relative entropy of 8.69 that lies closer to the experiment than the one calculated with the modified DFIRE potential (10.87). The p53 TRANSFAC matrix has been compiled from 17 SELEX binding sites and has a corresponding lower entropy (1.72) than the other two experimental ones.

**Figure 3:**
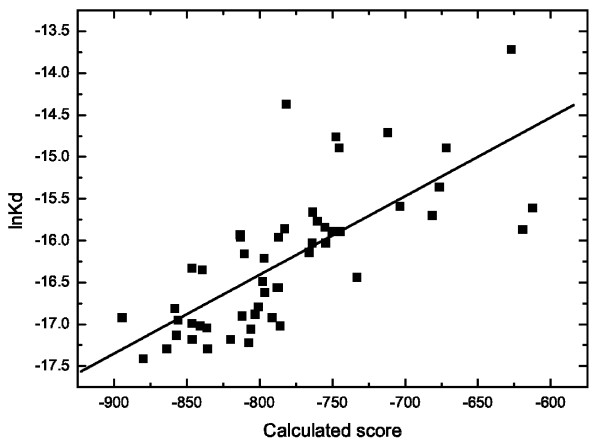
**The calculated scores (according to Eq.1) of the 51 oligonucleotides provided in Ref. **[18]** plotted against the logarithms of the dissociation constants measured in the same study**.

There are few experimental studies that focused extensively on measuring the p53-DNA binding affinity and from these we selected the two largest independent data sets, published in Ref. [18] and Ref. [20]. We calculated the TF-DNA binding energy scores of the altogether 51 oligonucleotide sequences published in Ref. [18] taking as a reference sequence number 600 in Table one from the corresponding work. Figure 3 shows the TF-DNA energy scores calculated by us plotted against the experimental lnK_*d *_shifts of the particular oligonucleotides with respect to the consensus sequence. There is a linear correlation between the experimental affinities and the calculated free binding energies (p value = 1.0E-9, R^2 ^= 0.54), the small deviations from the regression line observed in the upper right part of the figure are most probably related to larger experimental errors observed at weaker TF-DNA binding. Another experimental work [20] investigated the binding of p53 to mouse DDB2 genes. Having taken the consensus sequences and corresponding dissociation constants provided in their paper we found a good agreement between experiment and theory, as suggested by the p value of the fit (2.0E-3) and the regression coefficient R^2 ^equal to 0.65. The oligonucleotide sequences, experimental lnK_*d *_shifts and the calculated by us binding energy scores for these two experimental sets can be found in Additional File 2.

### p53, p63 and p73 dimers

The p63 and p73 tumor proteins belong to the p53 transcription factor family, reaching 63% sequence identity to p53 in the DNA binding domain. Correspondingly, p53, p63 and p73 have overlapping and distinct functions - p53 regulates the stress response to suppress tumors; p63 is essential for ectoderm development; and p73 might regulate both stress response and development.

To the best of our knowledge, crystallographic structures of the DBDs of p63 and p73 complexed with DNA have not been resolved yet, however, solution structures of the C-terminal domains containing the corresponding DBDs are available (PDB entries 1RG6 and 1COK)). As these three proteins belong to the same family and show high DBD sequence similarity, we used homology modeling in order to reconstruct the complete p63 and p73 DBDs using the p53 DBD as a template. We compared the performance of the web servers ESyPred3D [21], Geno3D [22], 3D-JIGSAW [23], Phyre [24], and SwissModel [25] in reconstructing the p63 and p73 DBDs using the p53 DBD. Further, considering the high sequence similarity of p63 and p73 to p53 and possibly the same binding mode in most of the cases, we aligned the modeled structures of p63 and p73 to the crystallographic structure of the p53 DNA binding domain in its homodimeric form (PDB code 2GEQ). Both p63 and p73 DBDs were modeled using as a template the p53 structure with PDB accession number 1GZH whose chain A had 55% sequence similarity to both p63 and p73. Although chain D from a p53-DNA complex (PDB entry 2AC0) showed higher similarity to p73 (62%), it included a smaller number of residues, therefore the former template was preferred.

The p63 and p73 DBDs were iteratively aligned to the p53 domain using the Pymol software package [26]. We chose the p63 and p73 DBD structures modeled with the SwissModel [25] server as they showed the smallest RMSD from the p53 domain - the alignment of the common C^*α *^atoms of p63 to those in the corresponding p53 domain showed an average RMSD of 0.45Å, for p73 it was about 0.44Å. The other web servers (ESyPred3D, Geno3D, 3D-JIGSAW, and Phyre) allowed also for modeling of chain flexibility in the protein structures using rotamer libraries, which led to very large RMSD values (up to 3Å) superposing the new DBDs to the p53 DBD. However, these servers provided structures that could be effectively used in protocols sensitive to chain flexibility, such as molecular dynamics studies and docking.

**Figure 4:**
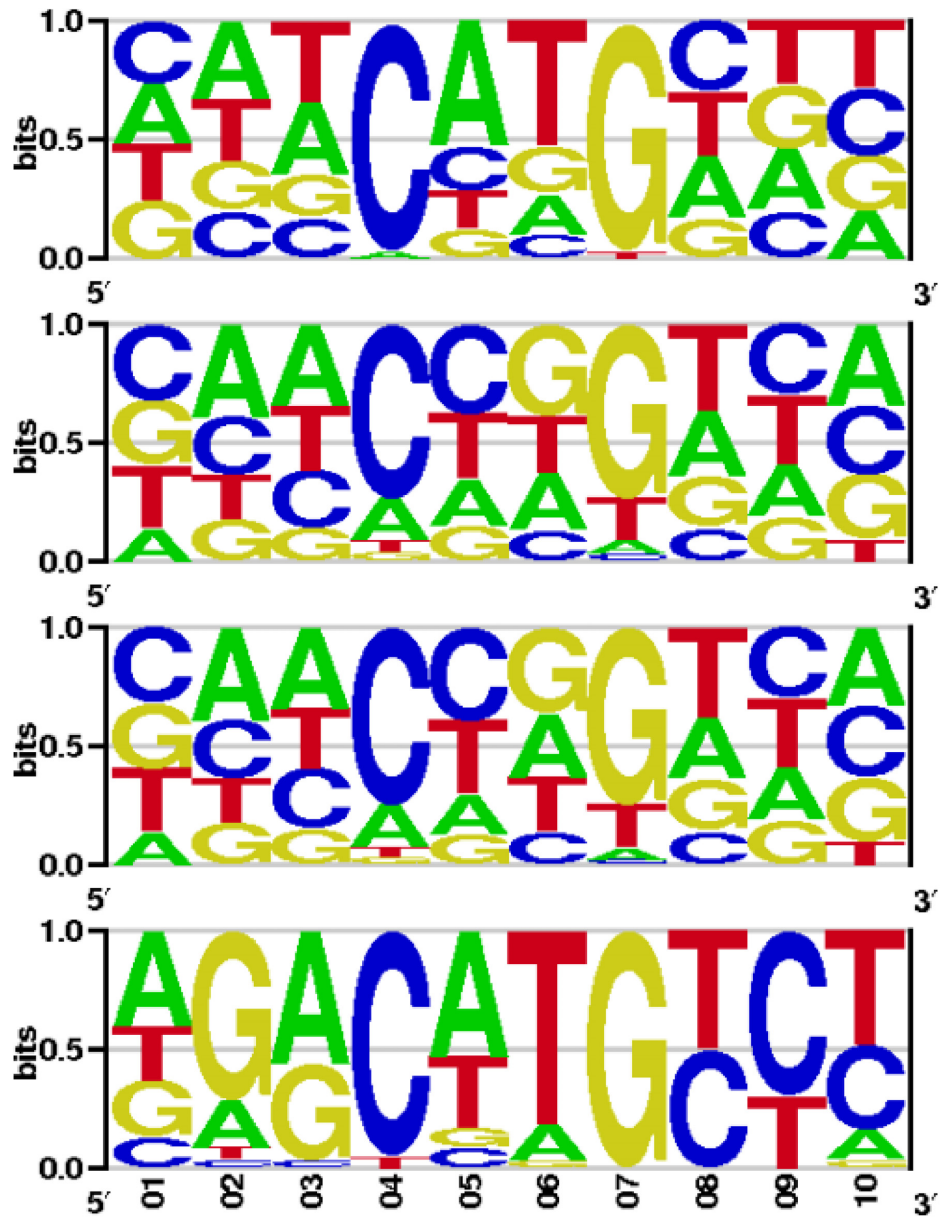
**Homology modeling using the p63 and p73 DNA-binding domains**. From top: the p53, p63, and p73 dimer PWMs from this study and the corresponding p53 TRANSFAC logo from entry V$P53_02. The p63 and p73 PWMs were obtained by homology modeling using the p53 binding domain. For detailed presentations, the logos were computed by plotting the frequencies and not by calculating relative entropy as in Fig. 2.

Finally, the aligned complexes were locally relaxed with AmberTools [27] in order to remove possible sterical clashes. The homodimeric p53, p63, and p73 PWMs were calculated using the scoring scheme presented above. The corresponding sequence logos of the three PWMs are shown in Figure 4 together with the logo obtained from the TRANSFAC matrix with entry V$P53_02. For comparison we calculated also the PWMs of the possible tetrameric complexes (not shown). In those calculations the p53 tetramer structure with PDB entry 2AC0 was used as a template, the results were well comparable to those obtained with the dimer complexes.

### Transcriptional targets of the p53 family

In order to quantify how similar were the newly computed homodimeric p53, p63, and p73 PWMs we decided to evaluate their performance on upstream sequences of genes known to be regulated by these factors. In TRANSFAC there are only few annotated p53 response elements mapped to promoters, which was not enough for comprehensive statistics. Hence, we selected a set of 85 genes which are known to be upregulated by p53 and/or p73. All promoters of a given gene were included in the scan with Match (altogether 126 promoters). We used three promoter windows of different length in accordance to the genomic coordinates provided in TRANSPRO. The smallest promoter window spanned [-900, 100] bp from the transcription start site (··TSS··), the other two had coordinates of [-1900, 100] bp and [-4900, 100] bp relative to the TSS. There were no overlapping promoters in the promoter window [-900,100] bp, 2 partially overlapping promoters were found in the [-1900,100] bp window, and 22 in the largest [-4900, 100] bp promoter window. However, we do not how the potential binding sites are distributed in the promoters and how many they could be, therefore we assumed that each promoter contained at least one site and scanned the whole length of all promoters.

**Figure 5:**
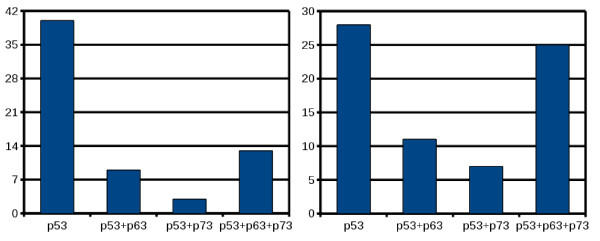
**Results of Match scan on 126 human promoter sequences**. On the left is shown the distribution of reported hits for the p53, p63, and p73 PWMs when the promoter window is set to [-1900,100] in respect to the transcription start site, on the right the same results using larger promoter window [-4900,100]. About the half of the p63 and p73 hits lay beyond the 2 kb promoter window.

Matrix cutoffs were calibrated to give an empirical prediction rate of (less than or equal to) 1 site in 10 K residues (p value of 1.0E-4). In order to take properties of real promoter sequences, such as repeats, TFBSs, or CpG-rich regions, into account, we randomly sampled a set of 126 human promoter sequences and selected a score threshold that satisfied the desired site frequency based on Match predictions. This procedure was repeated several times which lead to insignificant changes in the matrix cutoffs. Then the promoter sequences were scanned with Match using the matrix cutoffs calibrated on the background sets. Figure 5 shows the fraction of promoters in which only potential p53-responsive elements have been predicted with Match, those with p53 and p63 hits, with p53 and p73 hits, and with hits of all three PWMs, respectively. In contrast, p63- or p73-only promoters seem to be relatively rare as Match returned only five promoters with p63 and p73 but no p53 hits in the promoter window [-1900, 100] bp (not shown in the Figure). In those five promoters (belonging to the DHRS3, FEN1, JAG2, SERPINA1, and TRIM32 genes) the two p63 and p73 binding sites reported in DHS3 overlapped, two out of four hits overlapped in FEN1, from the altogether five hits in JAG2 only two overlapped, and the two p63 and p73 hits in SERPINA did not overlap. TRIM32 was reported as a p63-only promoter (having two p63 hits) at matrix cutoff corresponding to p value of 1.0E-4.

Restricting the promoter window to [-900, 100] bp in respect to the gene TSS produced many p53-only hits (results not shown) but also many non p53-hits in particular promoter sequences. p63-only sites were reported in altogether eight promoters belonging to eight genes (CTNN31, GDF15, IL4R, KLHL21, MDM2, RAD17, SERPINA1, TRIM32). The p63 sites lay 715 bp, 10 bp, 474 bp, 725 bp, 415 bp, 289 bp, 42 bp, and 702 bp before the particular TSS, respectively. For these particular 8 promoters, it means that the p63 sites lie closer to the TSS (could be found in the [-900,100] bp window), but potential p53-binding sites were not found in this small promoter window. Here, from the 8 p63-only promoters found in the [-900, 100] bp window, only two (SERPINA1 and TRIM32) did not contain potential p53 hits when extending the promoter window to [-1900, 100] bp. At the small promoter window [-900, 100] bp Match returned p63 and p73 hits in altogether 7 promoter sequences belonging to the CDC42RP2, DHRS3, FEN1, GRK5, JAG2, p53AIP1, and TP53 genes. The reported p63 and p73 sites overlapped completely in the CDC42RP2, DHRS3, GRK5, and TP53 promoters and partially in the other three promoters. In one of the JAG2 promoters the p63 site lay 782 bp away from the p73 site, in FEN1 and p53AIP1 the separation amounted to 170 bp and 361 bp, respectively. On the other hand, in the third JAG2 promoter the suggested p63 and p73 sites lay next to each other. In some of the cases the reported p63 and p73 sites overlapped, however, several p63-only promoters were detected. None of the p53 hits returned by Match overlapped with p63 or p73 ones.

In summary, the results obtained with Match suggest that the three new structure-based matrices return unique hits. Particular p63 sites reported in promoters of the CAMLG, COL5A2, PPL, and SYNE2 genes lay about 50 bp or less from the proposed p53 binding sites. Similar to these results, the p73 sites reported in promoters of the GDF15, FXR1, and TSPAN1 genes lay between 17 and 42 bp away from p53 sites.

Concluding this section, the results obtained with the three newly computed PWMs suggest that the p53 transcriptional activity could be regulated by the other two family members. The promoter sequences used here are available in Additional File 3 together with the corresponding list of TSS genomic coordinates (NCBI 36/human genome build 18).

### NF-*κ*B

**Figure 6:**
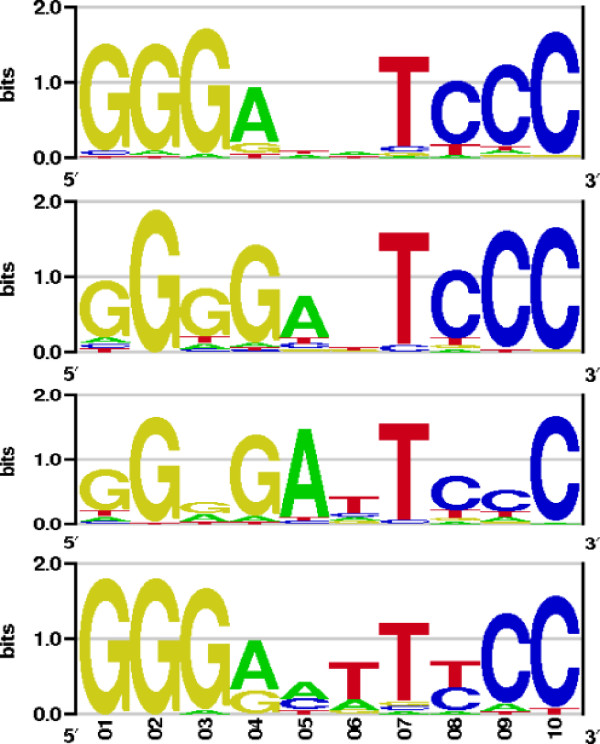
**From top: the p50 homodimer, p50p65, and p50RelB heterodimers, and the general NFKB logo from the TRANSFAC matrix V$NFKAPPAB_01**.

The good correlation between experimental affinity data and predicted TF-DNA free binding energies shown in Figure 3 and Additional File 2 encouraged us to calculate PWMs of transcription factors from other structural classes. We further chose the NF-*κ*B family for which experimentally measured dissociation constants are available to which we compared the binding energy scores computed here. We calculated PWMs for three members of the NF-*κ*B family: the p50 homodimer, the p50RelB and the p50p65 heterodimers. Sequence logos of these three PWMs together with a general logo from the corresponding TRANSFAC entry V$NFKAPPAB_01 are shown in Figure 6. The relative entropies of these members of the NF-*κ*B family calculated with Eq.4 are as follows: 2.65 for p50p50, 2.38 for p50p65, 2.84 for p50RelB, and 2.29 for the corresponding TRANSFAC PWM. The three newly computed matrices presented here differ slightly from each other, but in general they are in a good qualitative and quantitative agreement with the general sequence logo from TRANSFAC. Comparing the matrices on the basis of relative entropy we would suggest that the p50p65 PWM computed from structure is the one closest to the experimental one. The left parts of the p50RelB and p50p65 PWMs are somehow similar to each other, as guanine is preferred at the fourth position in the corresponding sequence logos, which is not the case for the p50 homodimer and the TRANSFAC PWM. On the other hand, the first two logos show similar CCC probabilities in their right parts. We also recalculated the p50p65 PWMs using the DDNA2 server, its PWM (shown in Additional File 1) had higher relative entropy (3.28) comparing to the experimental PWM (2.29) and the other NF-*κ*B matrices we obtained.

We also addressed the quality of the structure-based matrices by scanning human promoters containing known, experimentally verified NF-*κ*B binding sites. We selected 69 human genes for which it is known that they are regulated by NF-*κ*B. The full sequences (each of length 11 kb) of the altogether 124 promoters belonging to these genes were extracted from TRANSPRO and are available in Additional File 4. Experimentally confirmed NF-*κ*B binding sites are found in only 31 out of the 124 promoter sequences belonging to 25 genes. All relevant experimentally verified sites (also from other TFs) in these 124 promoters are listed in Additional File 5, 58 of them are NF-*κ*B response elements. As TRANSFAC provided no matrix compiled particularly for the RelB protein we excluded the newly computed p50RelB PWM from the scan. Consequently, we compared the performance of the new p50 homodimer and the p50p65 heterodimer PWMs to two of the newest matrices of the TRANSFAC library, V$P50P50_Q3 and V$P50RELAP65_Q5_01, which were built from homodimeric and heterodimeric response elements, respectively. Score cutoffs were calibrated on randomly sampled background promoter sequences in the same way as for the p53 family members. We worked again with matrix precision corresponding to a p value of 1.0E-4. We assumed that each promoter contained at least one site and scanned the whole promoter length.

A matrix scan with Match was performed on 31 promoter sequences containing 58 experimentally confirmed binding sites. Here, the alternative promoters of four genes (CCL5, IFNG, IL2, and MMP9) partially overlapped. The newly computed p50p50 and p50p65 PWMs detected 30 and 25 sites, respectively. The TRANSFAC p50p50 PWM recovered 25 sites, whereas 26 response elements were reported by the TRANSFAC p50p65 PWM. Binding site locations of the p50p50 motifs differed in 8 sites, of which three sites were only recovered by the TRANSFAC p50p50 PWM. In the comparison of p50p65 motifs, the structure-based matrix reported 5 sites missed by the TRANSFAC PWM, however 6 matches of the TRANSFAC matrix were not detected by our PWM. Comparing the two groups, the newly computed p50p50 PWM discovered 7 sites that the p50p65 PWM did not find, but missed 2 which were covered by the p50p65 PWM. The p50p50 TRANSFAC PWM discovered 4 sites different from those reported by the TRANSFAC p50p65 PWM, but missed 5 sites detected by the latter PWM.

In summary, the PWMs computed from crystal structures performed better than the TRANSFAC ones recovering altogether 55 sites, while both TRANSFAC PWMs yielded 51 hits. However, there are two factors that influence these results - for many of the experimental binding sites it is not known with which NF-*κ*B-family member (p50, p52, p65, RelB) they interact. Here, we focused on two very specific cases, namely p50 homodimer and p50p65 heterodimer, and omitted p52 and RelB. Second, we did not compare the PWMs presented here to all 6 other NF-*κ*B-related matrices annotated in TRANSFAC that could probably cover all 58 response elements found in the 31 promoters. The comparison shown here aimed at evaluating the quality of the matrices presented in this work.

### GABP and ER*α*

**Figure 7:**
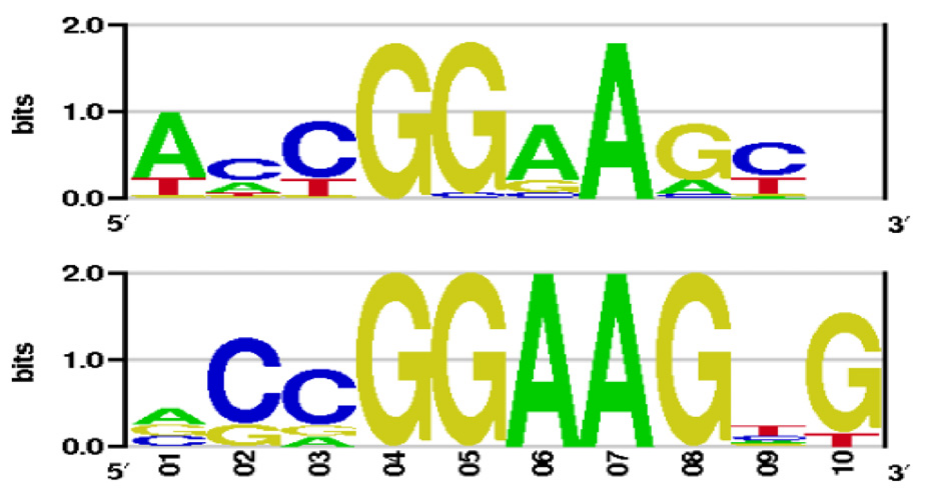
**The GABP heterodimer sequence logo from this work (top) and the corresponding logo from TRANSFAC entry V$GABP_B (bottom)**.

As representatives from other structural TF classes we chose the GABP heterodimer and the ER*α *nuclear hormone receptor whose family members are widely expressed in various eukaryotic genomes. The helix-turn-helix GA-binding protein (GABP) is unique among the ETS-family transcription factors as it functions as a heterodimer composed of an *α *and a *β *subunit. The *α *subunit, encoded by the GABPA gene, contains the ETS DNA-binding domain, while the *β *subunit, encoded by an unrelated gene, GABPB2, contains the transcriptional activation domain as well as four ankyrin repeats necessary for dimerization with the DNA-binding subunit. For the GABP matrix computation we used its heterodimeric complex, while for ER*α *its homodimer complexed with DNA was used. Figure 7 shows the newly obtained GABP matrix logo together with the corresponding logo from TRANSFAC (from entry V$GABP_B). The intensities of the first three nucleotides in the newly computed PWM compare well to those found in the corresponding TRANSFAC logo although the intensive guanine signal seen in the latter PWM vanishes in the atomistically modeled matrix. Nevertheless, the GG-repeat as well as adenine at the seventh position in the new matrix are also quantitatively well captured comparing to the corresponding PWM from TRANSFAC.

**Figure 8:**
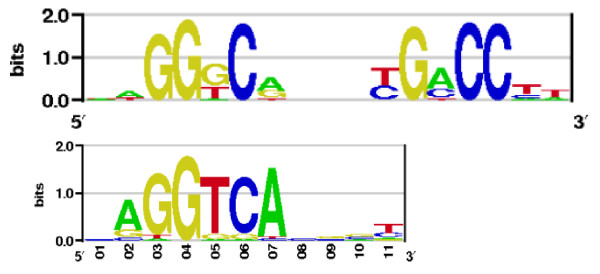
**The ER*α *logo from this work (top) and the corresponding logo from TRANSFAC entry V$ER_Q6_02**.

The ER*α *estrogen receptor belongs to the family of Cys4 zinc finger proteins. Figure 8 shows the sequence logo of the homodimer and a half-site (monomer) logo from TRANSFAC entry V$ER_Q6_02. The GG-signal seems to be well reproduced by the statistical potential used here, and the AGGTCA consensus suggested by TRANSFAC is better reproduced in the right part of the PWM computed in this work. Thymine seems to compete with guanine in the fifth position of the structure-based matrix, while the TRANSFAC logo exhibits thymine dominance at that position. The all-atom matrix computation used here allows for discrepancies between two homodimeric binding sites as shown in Figure 8. Experimentally, it is more difficult to capture such structural details as most of the experiments use half-sites in the binding affinity measurements [18] and the PWMs derived from such size also have to be symmetric. Probably the strongest advantage of the method presented here over the experimental ones is the opportunity to compute PWMs from complicated TF structures like homo- and heterodimers, or tetramers, as in the experiment this is often difficult to account for.

## Discussion

### p53, p63, and p73 - similar but different

The computation of the p53, p63, and p73 PWMs raises the question if the matrices of the three transcription factors could report unique response elements. A recent ChIP-Chip study [28] discovered about 5800 target sites for p63 across the human genome. It was suggested that p63 may regulate its own expression as well as crossregulate expression of p53 and p73 as it bound all members of the p53 gene family. Interestingly, a strong overlap between p63 and p73 sites was found [29], as nearly 80% of the altogether 488 p73 sites overlapped with p63 ones detected under the same experimental conditions. The authors identified also 327 high-confidence binding sites for p53, 62 of them overlapping with p63 sites. According to our results obtained with Match at least 80.5% of the p63 and p73 hits overlap. On the other hand, most of the reported p53 sites did not overlap with those of p63 and p73. The fact that many of the best-scoring p63 and p73 binding sites lay at least 2 kb before the transcription start sites suggests that indirect regulation of p53 by p63 or p73 is possible.

### The NF-*κ*B family - comparison to other studies

Experimental TF-DNA binding energy data for p50 and p65 provided in the PRONIT [30] database were not sufficient for an extensive comparison with our results. After having discarded repeating binding DNA sequences we obtained only five independent binding energies ΔG that provided a linear fit to our results (not shown). The gel electrophoresis study by Matthews and coworkers [31] on the p50 homodimer provided altogether nine different sequences and relative molarities *M *at 50% binding. The experimental results correlated well with the calculated binding energies, giving a regression coefficient of 0.75 and a p value of 2.0E-3 (data shown in Additional File 2). We also found a good agreement between calculated energies and experimental binding affinities derived from microarray data as presented by Wang et al. [32]. This group investigated the binding affinities of the p50 homodimer to the wild type and single-nucleotide mutant Ig-*κ*B sites with a dsDNA microarray produced with a novel scheme. The binding energy scores we calculated for the altogether thirty-one 10 bp target sequences plotted against the measured fluorescence intensities (Additional File 2) provided a good qualitative agreement (p value = 4.1E-5, R^2 ^= 0.45).

### Estimation of true and false positives

In order to quantify the performance of the PWMs in distinguishing true positive from false positive hits we prepared corresponding positive and negative sets, the positive sets are available in Additional File 6. As a comprehensive collection of p53-bound sequences we used the ChIP-Pet data presented in Ref. [33] assuming that each fragment contains a p53 response element. After discarding the largest fragments (> 2000 bp) we had a set of 512 positive fragments that we extended to 2000 bp each in order to avoid biases arising from different fragment lengths. For creation of negative sets we randomly sampled nonoverlapping human promoters and took for each of them a promoter window of [-1900,100], so that the fragment length was equal to 2000 bp. All negative sets had same number of fragments of the same lengths as compiled in the corresponding positive set, where for each PWM group to be compared, p53, NF-*κ*B, GABP, and ER*α*, different background sets were generated. In this way, altogether 10 different negative sets for a group were prepared. Regarding the evaluation of the NF-*κ*B PWMs we were not successful in finding a comprehensive ChIP-Chip set and used the 124 promoter sequences studied above assuming that each of them contains at least one NF-*κ*B binding site. For GABP and ER*α *we used ChIP-Seq data from Ref. [34] and [35]. From each of the two ChIP-Seq sets we selected 500 fragments that were extended to 2000 bp length. The receiver-operator characteristic (ROC) was analyzed by iterating over all PWM scores (0.000,1.000) and calculating proportions of recognized 2 kbp-sequences in positive and negative sets, we sampled the average area under the curve (AUC) over the ten negative sets.

**Figure 9:**
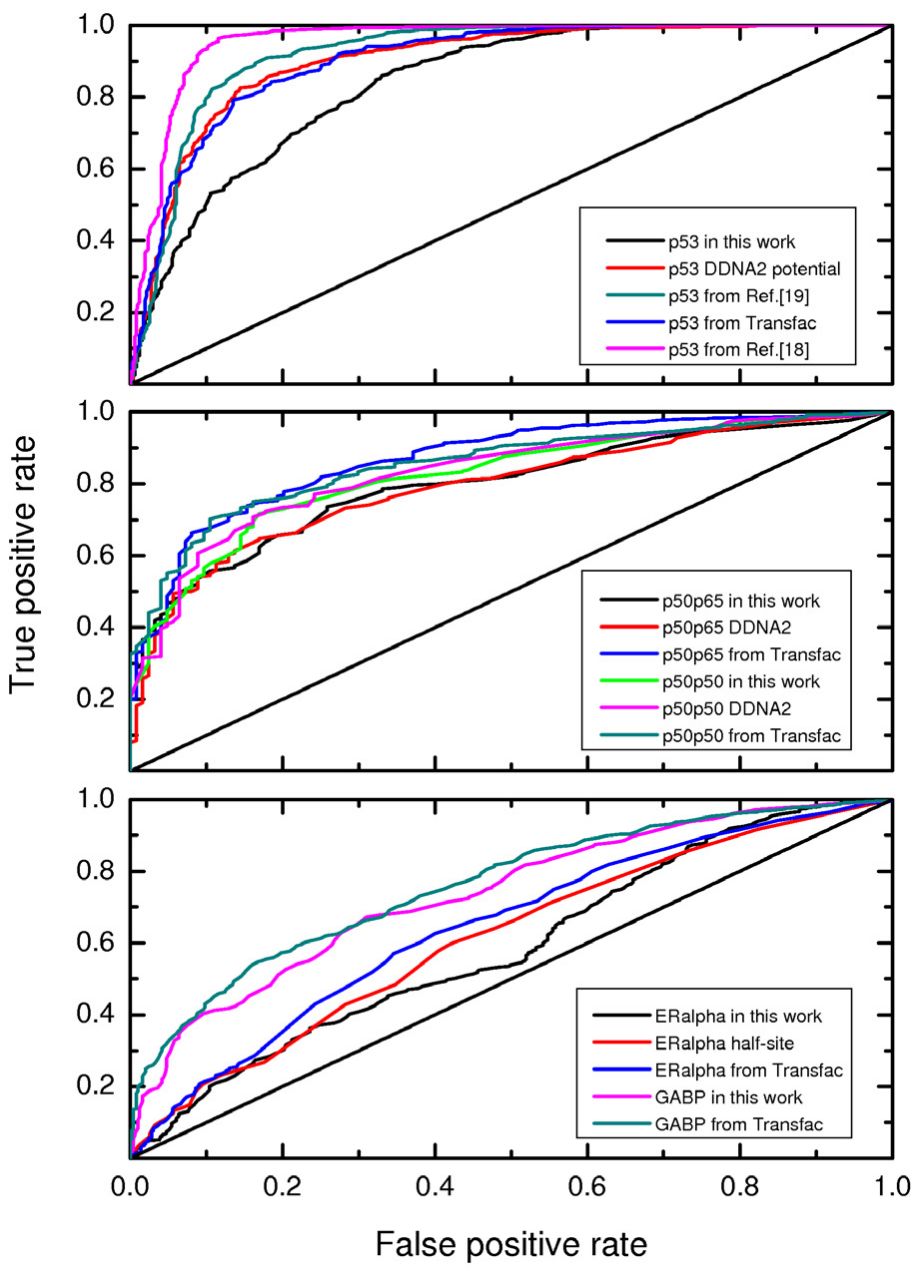
**Estimation of the PWM accuracy in distinguishing true positive from false positive TF binding sites**. From top to bottom: performance of the p53 tetramer, NF-*κ*B, GABP and ER*α *PWMs.

Figure 9 shows true positive rates (TPR) and false positive rates (FPR) for the p53 tetramer (top), NF-*κ*B (middle) and GABP and ER*α *(bottom panel). Regarding the area under the curves the p53 PWMs performed best, with the p53 PWM from Ref. [18] having the largest AUC of 0.956 ± 0.002 followed by the PWM from Ref. [19] compiled from 100 experimentally verified p53 response elements, having AUC of 0.921 ± 0.003. The p53 PWM recalculated with the DDNA2 server and the corresponding TRANSFAC matrix performed quite comparable (AUC of 0.901 ± 0.007 and 0.902 ± 0.006, respectively). In this Figure, the p53 PWM developed here showed somehow smaller AUC of 0.841 ± 0.004 comparing to the other PWMs.

The sensitivity of the NF-*κ*B PWMs is obviously lower in comparison to the performance of the p53 tetramer matrices, which could be also connected to the fact that the length of the NF-*κ*B response element amounts half of the p53 tetramer one. Within some small deviations all NF-*κ*B PWMs performed comparably well in distinguishing true from false binding sites. The AUC calculation revealed that the NF-*κ*B TRANSFAC PWMs performed minimally better than the theoretically calculated ones, p50p50 from TRANSFAC yielding AUC of 0.855 ± 0.018, p50p65 from the same source had AUC of 0.863 ± 0.018. The AUC values of the theoretically derived PWMs were as follows: 0.825 ± 0.019 for the p50p50 PWM and 0.798 ± 0.032 for p50p65 PWM computed here, 0.833 ± 0.016 for the p50p50 PWM and 0.792 ± 0.037 for the p50p65 PWM recalculated with the DDNA2 server.

We also evaluated the performance of the structure-based p50p50 and p50p65 PWMs and their TRANSFAC homologs using 45 sequences of experimentally verified response elements that did not overlap with the 58 binding sites found in the 124 NF-*κ*B-regulated promoters discussed above. The DNA fragments used in SELEX and gel-shift experiments are typically very short (~30 bp), therefore we had to generate random DNA sequences of the same length instead of extracting such pieces from promoters, as the latter contain binding sites and repeats that could bias the statistics. We sampled AUC over 10 background sets, the ROC plot is shown in Additional File 7. In this case, both p50p50 PWMs showed better performance than the p50p65 ones, the p50p50 PWM computed here having AUC of 0.772 ± 0.030, the p50p50 from TRANSFAC having AUC of 0.779 ± 0.027, while the p50p65 PWM from this work and the corresponding TRANSFAC one had smaller AUCs (0.701 ± 0.069 and 0.725 ± 0.045, respectively).

Further, both GABP PWMs performed comparably well, the TRANSFAC matrix slightly outperforming the newly developed one (AUCs of 0.759 ± 0.009 for the TRANSFAC PWM and 0.735 ± 0.009 for the one presented here). Analyzing the sequence logo in Figure 7 one finds that adenine in position 6 in the newly computed matrix is not so dominant as in the TRANSFAC one, certainly the quality of the crystal structures influences the sensitivity of the corresponding PWMs.

Finally, we compared the performance of the homodimeric ER*α *PWM computed here and a monomeric one (half-site) using the right side of the sequence logo shown in Figure 8, while a corresponding TRANSFAC matrix is available only for a half-site of the response element. To our surprise, none of these three PWMs performed really well comparing to the p53 and NF-*κ*B results. The homodimeric PWM computed here delivered an AUC of 0.588 ± 0.012, the half-site PWM had an AUC of 0.613 ± 0.012, the TRANSFAC half-site PWM performed slightly better producing an AUC of 0.643 ± 0.017.

In summary, our PWMs were not as good as the best matrices compared in this study on the basis of their ability to distinguish true positive from false positive sites. As the p53 PWM from Ref [18] was computed from affinity measurements, the PWM from Ref [19] compiled from 100 response elements, and the TRANSFAC matrix from 17 SELEX fragments, it is clear that these matrices rely mostly on strong binding sites, which is not the case with the PWMs computed in the present work. Nevertheless, the performance of the newly computed p53 PWM was comparable to that of the experimentally derived PWMs, while its computation was highly effective regarding time and costs.

All NF-*κ*B PWMs presented here showed similar performance regarding the positive set consisting of 124 promoters. The scan on the smaller set containing 45 response elements showed that both p50p50 PWMs performed better than the p50p65 ones. Interestingly, although both p50p50 PWMs had similar AUC values, the p50p50 matrix computed from structure recovered 30 out of the 58 NF-*κ*B binding sites found in the 124 promoters, while the TRANSFAC one detected 25 sites.

While the p53 and NF-*κ*B matrices performed well in distinguishing true positive from false positive sites, the GABP and especially the ER*α *PWMs discovered also many false positives. One possible reason could be the use of fragments computed from ChIP-Seq experiment as positive sets, as it is known that not all detected peaks contain binding consensus of the factor [36].

### Limitations and advantages of the model

#### Quality of the crystal structures

As discussed in Ref. [3], the greatest weakness of the used potential is the lack of local structural detail such as presence of hydrogen atoms in the scoring function. Another and larger source of errors are the crystal structures we used to assess the effect of base pair mutations and subsequent calculations of the binding energies. The quality and reliability of the computed free binding energies are strongly dependent on the resolution of the crystal structures. One possible improvement could be the introduction of a term accounting for the number of formed hydrogen bonds as well as their directions, as the present potential does not consider them. Another area of improvement could be considering the heavy atom types as protein class-specific, for example one set of atom types for leucine zipper proteins, other types for helix-turn-helix proteins, etc. In this way, more precise and also protein class-specific PWMs could be constructed.

#### Interdependent positions in the binding sites

PWMs are calculated under the assumption that residues in different positions in the matrix contribute independently from each other to the total binding strength. If the binding sites contain interdependent positions, a richer model than a PWM is required to adequately capture the dependencies, which typically requires more training data. Just at this point lies a potential strength of the structure-based approach, as different base pair combinations can be tested fast and cheaply in-silico. Hence, application of our approach to more sophisticated models for transcription factor binding sites presents an important direction for further research [37]. A nice example of palindromic sequence coupling has been studied in Ref. [19] on the basis of base inversion correlations. The Molecular Dynamics techniques could be also a suitable choice in this case, as one could insert double or triple mutations simultaneously in the DNA chain, then compute the free binding energy with a custom function.

#### Deformed DNA structures in the complexes

When the protein and/or DNA structure deforms significantly upon binding, the potential used in this study will most probably perform poorly, as it has been calibrated against crystallographic data containing native interatomic coordinates. The mutation scripts we used work with B-DNA as they use precompiled rotamer libraries that are needed in order to reproduce the native geometry (avoid clashes) upon residue mutation. If the DNA chain is deformed, one possible solution of this problem is the use of docking allowing for chain flexibility. Such approach is used by the HADDOCK server [38] that utilizes information from identified or predicted protein interfaces in ambiguous interaction restraints to drive the docking process. Once a reasonable protein-DNA configuration has been identified, one could proceed to the PWM computational procedure using the potential applied here. Alternatively, a short initial optimization (100-300 molecular mechanics steps) of the protein-DNA complex could be performed using a software like AmberTools, which would remove atomic contacts of strongly deviating length.

## Conclusions

In the present work a statistical potential was applied for PWM calculations using a novel scoring scheme for estimation of protein-DNA free binding energy. In contrast to the computationally intensive molecular dynamics techniques the model presented here combines efficiency and accuracy by modeling both transcription factor and DNA chain atomistically. The computational workflow aims to cover all (4^*N*^) possible nucleotide combinations. Our first goal was to check the efficacy of the potential against well studied TFs like p53, for which sufficient experimental data for comparison is available. The p53 tetramer PWM computed here correlated very well with a newly published one [19] compiled from 100 response elements.

The PWMs of p63 and p73 were computed using homology modeling and a structure of the DNA-binding domain of the p53 dimer as a template. Having performed a promoter scan on 85 p53/p73-regulated human genes we found that about half of the p63 and p73 hits reported by the Match algorithm lay more than 2 kb upstream of the corresponding transcription start sites. In most of the cases the best-scoring p63 and p73 binding sites did not overlap with the p53 ones, which suggests that p63 and p73 could regulate the p53 transcriptional activity.

The structure-based PWMs of the NF-*κ*B family members p50p50 and p50p65 performed comparably to the corresponding TRANSFAC ones in distinguishing true positive from false positive sites. Performing a Match scan on experimentally verified NF-*κ*B response elements we found that the newly computed p50p50 PWM recovered 5 more experimental binding sites than the corresponding TRANSFAC matrix.

While the general idea of deriving position weight matrices from known protein-DNA structures has been explored before [4-6,17], this work, besides providing a new method for PWM calculation from 3D structures, is the first that systematically compared structure-based matrices with those derived through traditional approaches. The proposed computational scheme used in combination with homology modeling can be successfully applied in PWM computations where crystallographic structures of the protein-DNA complexes are not available, which is the case for many transcription factors. Taking as an example the p53 family, when the binding mode is known and a crystallographic structure of a member of the family complexed with DNA is available, NMR structures of the particular protein DNA-binding domains can be used as templates for homology modeling and PWM computations. In this way, PWMs of new or little-known transcription factors can be accurately determined avoiding time and resource consumming affinity measurements.

## Authors' contributions

DA and PS designed the algorithm, DA conducted the calculations and wrote the manuscript. AK performed the promoter scan and helped to draft the manuscript. All authors read and approved the final manuscript.

## Supplementary Material

Additional file 1 **All PWMs used in this work, both computed and from literature**. All Matrices. Click here for file

Additional file 2 **Oligonucleotide sequences from literature, their experimental binding affinity values and calculated affinity scores**. Binding Affinities. Click here for file

Additional file 3 **The promoter sequences of the p53-regulated genes used in this work**. Included are promoter windows of [-900, 100] bp, [-1900, 100] bp, and [-4900, 100] bp in respect to the promoter TSS. Click here for file

Additional file 4 **124 NF-*κ*B-regulated promoter sequences used for validation of the PWM model**. The promoter windows span over [-10000, 1000] bp in respect to the TSS, the length of each promoter sequence is 11 kb. The genomic coordinates of the particular TSS are listed in Additional File 5. Click here for file

Additional file 5 **A list of experimentally verified TF binding sites for the 124 NF-*κ*B-regulated promoters.** Output from ExPlain 3.0. Gene names are presented in the first column of the table, the 5' most promoters are marked with '_1' in the second column, TSS starts(NCBI 36/hg18) are listed in the 3^*rd *^column. The promoter coordinates of the response elements are provided in the 5^*th *^column, the name legend in the 4^*th *^column. Coordinates are provided starting from one in respect to the promoter window [-10000, 1000]. The distances provided in the 4^*th*^column are calculated in respect to virtual TSSs and are not relevant for comparison. Click here for file

Additional file 6 **All positive sequence sets used for calculation of the true positive/false positive rates as presented in **Figure 9. Chip chip sequences. Click here for file

Additional file 7 **True positive/false positive rates estimated for the homo- and heterodimeric NF-*κ*B PWMs using sequences of 45 experimentally verified response elements**. NFKB EXP FP FN. Click here for file
