# Supplementary figures and images for: Creating PWMs of transcription factors using 3D structure-based computation of protein-DNA free binding energies
Source: BMC Bioinformatics. 2010 May 3;11:225. doi: 10.1186/1471-2105-11-225 (PMC2879287; doi:10.1186/1471-2105-11-225)

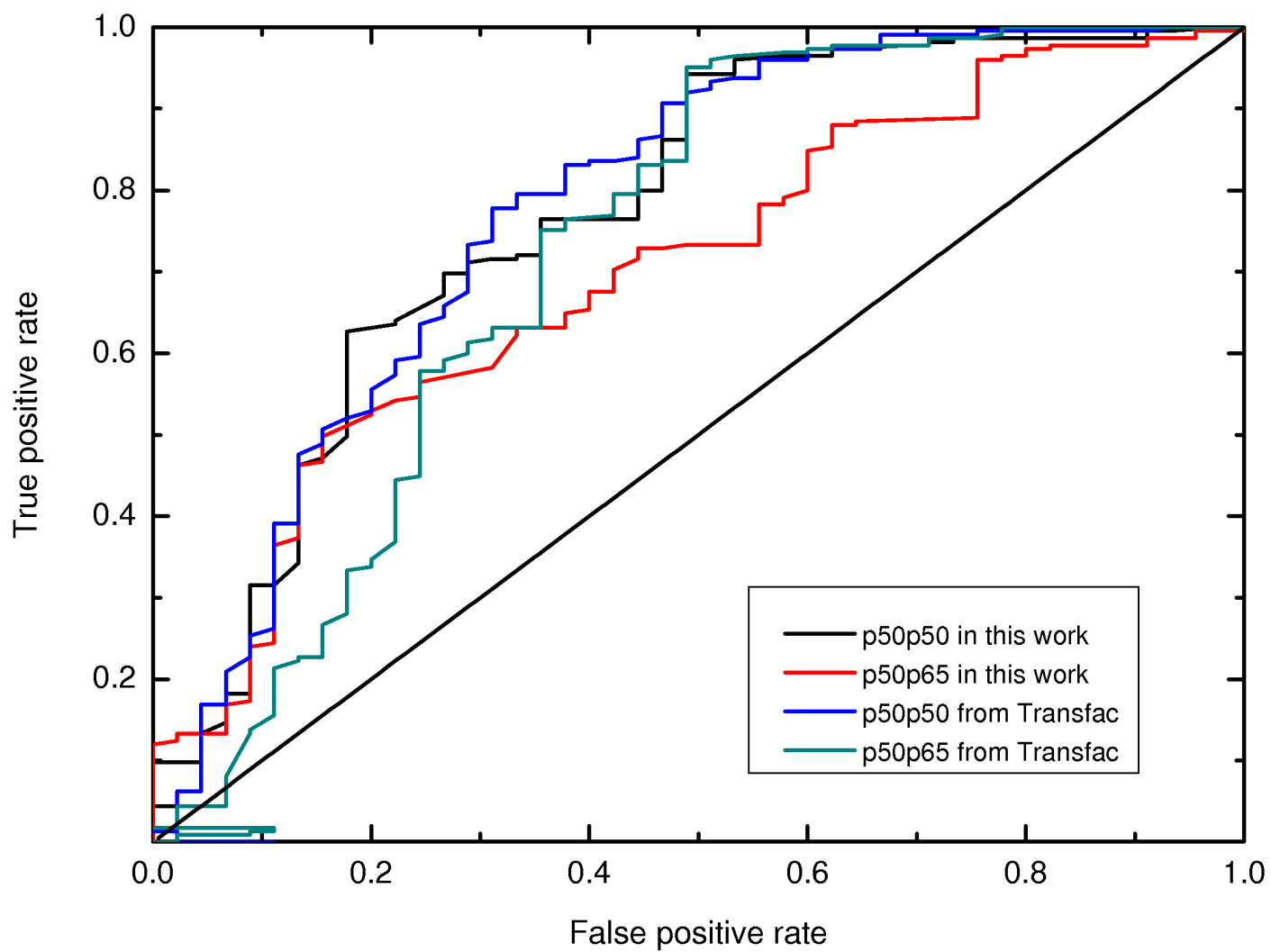

Supplement: Additional file 7 — True positive/false positive rates estimated for the homo- and heterodimeric NF-κB PWMs using sequences of 45 experimentally verified response elements. NFKB EXP FP FN. [file 1471-2105-11-225-S7.PDF]
